# Associations between dietary patterns and an array of inflammation biomarkers and plasma lipid profile in postmenopausal women

**DOI:** 10.1186/s12905-023-02417-w

**Published:** 2023-05-12

**Authors:** Bolaji L. Ilesanmi-Oyelere, Marlena C. Kruger

**Affiliations:** 1 School of Health Sciences, College of Health, Massey University, Palmerston North, New Zealand; 2 Human Nutrition and Dietetics Department, College of Health Sciences, Abu Dhabi University, Abu Dhabi, United Arab Emirates

**Keywords:** Dietary patterns, Food intake, Nutrient intake, Inflammation markers, Cytokines, Lipids

## Abstract

**Objective and design:**

In this cross-sectional study, evaluation of the association between four dietary patterns, nutrients and food intakes and an array of systemic inflammation biomarkers and lipid profile among 80 New Zealand postmenopausal women were conducted.

**Materials:**

Eighty postmenopausal women participated in the study. A validated food frequency questionnaire was used to collect nutrients and food intake. Four dietary patterns were identified by principal component analysis (PCA) and plasma samples collected for inflammatory biomarkers and lipid profile measures.

**Results:**

There were negative correlations between intake of dietary fibre, soluble and insoluble non-starch polysaccharides (NSP), vitamin C and niacin and with almost all the inflammatory markers for the whole group. Vegetables, tea/coffee and especially fruit intake were negatively correlated with the inflammatory biomarkers in the whole group. A high intake of Pattern 1 (potato, bread, and fruit pattern) was associated with a low risk of high interferon (IFN)-α2, IFN-λ, interleukin (IL)-6 and IL-8 levels while a high intake of Pattern 3 (fast-food pattern) was associated high risk of IFN-α2 levels. Multiple linear regression showed a negative correlation between Pattern 2 (soups and vegetables pattern) and levels of C-reactive protein (CRP) as well as ferritin. A positive association was observed between Pattern 3 (fast-food pattern) and CRP levels. Positive correlation was also observed between Pattern 2 and high-density lipoprotein (HDL) and total cholesterol (TC) levels, Pattern 4 (meat and vegetables pattern) was however negatively correlated with TC, low-density lipoprotein (LDL) and TC/HDL ratio.

**Conclusions:**

The result of this study reinforces the contribution and role of diet in modifying inflammation in postmenopausal women.

## Introduction

Chronic low-grade systemic inflammation has been associated with risk of developing many metabolic diseases of aging. Although inflammation is a crucial natural physiological process for health maintenance and recovery after an injury or infection, chronic low-grade inflammation has however been indicated as an important part of the pathogenesis of many chronic diseases of the aged population [1]. Chronic low-grade inflammation has been linked to inflammation-related diseases such as ischemic heart disease, stroke, bowel diseases, arthritis, cancer, diabetes mellitus, chronic kidney disease, non-alcoholic fatty liver disease (NAFLD) and autoimmune and neurodegenerative conditions [2]. Modifiable lifestyle factors such as diet and physical activity however has the potential to alleviate the complications and morbidity because of systemic inflammation. Postmenopausal women tend to lose estrogen levels from the onset of menopause. The hormone estrogen is known to be anti-inflammatory, therefore as the hormone decreases the inflammation levels increase indicating chronic systemic inflammation [3].

Diet has been proposed as an effective measure for reducing inflammation to alleviate the complications of the associated chronic disease. Dietary intake has been investigated in various types of ways in relation to low-grade inflammation. One approach includes focus on the overall dietary intake taking into account the trends in food components known as dietary patterns. Examples include population studies and intervention studies which have indicated that a Mediterranean [4], healthy [5], or prudent dietary pattern [6] may be beneficial in reducing inflammation. A second approach involve examining the role single foods plays in lowering inflammation. Diets high in fruits and vegetables have been associated with lower concentrations of circulating C-reactive protein (CRP) [7] and fibrinogen [8]. Thirdly, some research has emerged focusing on specific antioxidant nutrients and non-nutrient components of foods such as β-carotene [9], Se, vitamin C, E and their effect on inflammatory status as a measure of the CRP levels [10]. Likewise, non-nutritive polyphenolic components such as tannic acid and flavonoids that are present in plant-based foods such as tea, cocoa, red wine as well as fruits and vegetables contain antioxidant and anti-inflammatory properties [11, 12]. These compounds are important for the effect of diet on inflammation status and metabolic health.

In addition, diet and lifestyle contributes to the prevalence of dyslipidemia which in turn result in mortality due to incidence of cardiovascular diseases [13]. Studies have researched the relationship between dietary patterns and blood lipid profile and low density lipoprotein (LDL)-cholesterol and triglycerides has been associated with nutrient-poor diet and high density lipoprotein (HDL)-cholesterol was correlated with nutrient-dense diet [14]. Two main strategies are used to define dietary patterns i.e., the a priori (hypothesis-orientated approach) or the a posteriori (empirical approach). This study employed the latter to investigate its correlation with an array of inflammation biomarkers.

Cytokines as inflammatory biomarkers, in general, are often complicated to study due to their synergistic effects and their ability to affect or enhance each other’s secretion, for example, IL-1 and TNF-α [15]. However, the cytokine network is significant in the regulation of the immune cells (primarily lymphocytes and macrophages) where a natural balance is needed for the physiological and pathophysiological metabolic homeostasis [16]. Most research studies have investigated mainly CRP and a few other inflammatory markers.

However, the present study investigated the relationship between dietary patterns of food intake among New Zealand postmenopausal women and 15 inflammatory biomarkers. Studies assessing the diet-inflammation association in this age group is lacking for all these markers considering the fact that ageing is associated with an increase in inflammatory markers.

## Methods

### Study participants

G*Power software version 3.0.10 was used to calculate the sample size with 90% power and an alpha of 5% for the phase 1 of the “Bugs’n’Bones” clinical study of which phase 2 is a subset. Eighty postmenopausal women participated in the phase 2 of the “Bugs’n’Bones” clinical study that took place in the Human Nutrition Research Unit (HNRU) located on the Massey University, Palmerston North campus from June to December 2017. Advertisement took place all around Massey University campus, in a local newspaper (Whanganui Chronicle) and by a recruitment agency, Trial Facts (https://trialfacts.com/). Participants recruited and included in the study were healthy women at least 5 years postmenopause. Women with any systemic diseases such as diabetes and liver diseases were excluded. Exclusion criteria also included smoking, excessive alcohol intake and significant weight gain or weight loss (i.e., > 5%) in the past year.

Written informed consent was provided by all participants prior to the commencement of data collection. The “Bugs’n’Bones” clinical study is registered with the Australian New Zealand Clinical Trials Registry (ANZCTR) with the number ACTRN12617000802303 (31/05/2017). The study was carried out in accordance with the approval and recommendations of Massey University Human Ethics Committee Guidelines with the number; Massey University Human Ethics Committee: Southern A, Application 17/17.

### Anthropometric measures and blood analysis

Participants’ body weight was measured using the Detecto 437 eye-level weigh beam physician scale to the nearest 0.1 kg. Measurement of the standing height was conducted using a stadiometer to the nearest 0.1 cm with participants wearing light clothes and no shoes on. Body mass index (BMI) of participants were then calculated as weight in kg divided by height in meters squared. The waist circumference was measured around the abdominal area above the hipbones by using a non-stretchable measuring tape to the nearest 0.1 cm.

Fasting venous plasma blood samples were collected, centrifuged, and then stored frozen at -80 °C before analysis. BioLegend® LEGENDplex™ Multi-Analyte Flow Assay kit’s instructions were used to prepare cytokine assays and the Beckman Coulter’s Gallios flow cytometer was used for the cytokine measurements. Levels of 13 cytokines were quantified in plasma from the samples, namely, IL-1β, IFN-α2, IFN-λ, TNF-α, MCP-1, IL-6, IL-8, IL-10, IL-12p70, IL-17A, IL-18, IL-23 and IL-33. LEGENDplex™ Data Analysis Software version 8.0 was used to analyze the data from the flow cytometer. Plasma levels of CRP and ferritin were measured using the electrochemiluminescence immunoassay “ECLIA”.

### Nutrient, dietary intake and dietary pattern assessment

Validated FFQs are a gold standard method of collecting information on quantity and frequency of foods consumed retrospectively [17]. Dietary assessment tools are therefore necessary for identifying patterns of an individual's diet in relation to any health issues associated. A validated semi-quantitative food frequency questionnaire (FFQ) from the Nutrition Department, Massey University, New Zealand was used to assess participants' diets. Nutrients and food composition were assessed using the validated FFQ comprising of 108 food items, from which 16 food groups were created. The FFQ was used to collect information and data on participants' frequency of food intake and beverage intake. Portion size, recipes, food and beverage intake were entered into the Foodworks version 9 Xyris software which was used to analyze the participants' diet data and the food and nutrient analyses of intake were calculated in grams [18].

### Statistical analysis

Principal component analysis (PCA) is an acceptable exploratory factor analysis for dimension reduction of data sets, while varimax rotation enables an orthogonal i.e., uncorrelated factor that enables better interpretability. PCA was used to identify four dietary patterns using 16 food groups that were generated from a 108-item semi-quantitative FFQ.

Varimax orthogonal rotation with Kaiser normalization was performed to reduce correlations between factors and increase interpretability of the results. Kaiser–Meyer–Olkin measure of sampling adequacy was 0.6 while Bartlett's test of sphericity was significant (< 0.001).

The IBM SPSS version 25 (IBM Company, Armonk, NY, USA) was used for all the statistical analyses in this study. The dietary patterns consisted of food intakes such as dairy (all types of milk, cheese, and yogurt/cream), fruits, vegetables, oily fish, red meat, potato, bread etc. The four dietary patterns were then obtained from the dimension reduction of 16 food groups. Analysis of variance (ANOVA) was conducted for the tertiles of each dietary pattern by the participants’ characteristics. Correlations of selected nutrients and food intakes were conducted with all inflammatory markers. Binary logistic regression and multiple linear regression was then performed showing the relationship between the dietary patterns generated and inflammatory markers. Multiple linear regression was also conducted using the ‘Enter’ mode to show the association between CRP, ferritin, IL-18, MCP-1 and the lipid profile and with all the four dietary patterns generated from the FFQ. All data analyses are reported significant at *P* ≤ 0.05.

## Results

As is shown in Table 1, four dietary patterns were generated from the food groups. The four dietary patterns included pattern 1 (potato, bread, and fruit pattern), pattern 2 (soups and vegetables pattern), pattern 3 (fast-food pattern) and pattern 4 (meat and vegetables pattern).

**Table 1:** Factor loading matrix for dietary patterns

Food groups	Potato, bread, and fruit pattern (Pattern 1)	Soups and vegetables pattern (Pattern 2)	Fast-food pattern (Pattern 3)	Meat and vegetables pattern (Pattern 4)
Potato	0.711	-	-	-0.231
Bread	0.696	-0.217	-0.285	-
Fruit	0.661	-	0.153	-
Tin/dry fruit	0.528	-	-	0.180
Spreads	0.164	0.725	-	-
Dairy	0.267	-0.523	0.163	-0.104
Water, tap	-0.166	0.514	-	-0.174
Soup	0.111	0.510	-0.123	0.228
Nuts, mixed	0.397	0.264	-0.659	-
Pizzas/burgers	-	-	0.600	-
Rice/pasta	-	0.354	0.598	-
Oily fish	-0.136	0.225	-0.566	-
White meat	-0.305	-	-	0.636
Vegetables	0.112	0.446	-0.177	0.634
Sauces/dressings	0.115	-	0.515	0.584
Red meat	-	-0.458	-	0.560
Variance explained (%)	14.526	13.536	12.006	8.691
Eigenvalue	2.324	2.166	1.921	1.391

Loadings |< 0.10| are excluded for ease of interpretation; Positive loadings indicate positive association, and negative loadings indicate negative association with the dietary pattern

Table 2 shows the analysis of variance (ANOVA) of the participants characteristics such as age, weight, body mass index (BMI), waist circumference (WC), activity energy expenditure (AEE), energy intake and lipid profile by tertiles of dietary pattern. The dietary patterns were classified into three (3) tertiles T1 (low tertile), T2 and T3 (high tertile).

**Table 2:** Participants’ characteristics and lipid profile by dietary pattern (Whole group)

	**Pattern 1**					**Pattern 2**		
	**T1 (low)**	**T2**	**T3 (high)**	***P *for trend**	**T1 (low)**	**T2**	**T3 (high)**	***P *for trend**
**Age (years)**	63.50	61.73	65.00	**0.034**	63.27	63.27	63.74	0.913
**Weight (kg)**	68.92	65.67	65.82	0.448	67.60	69.48	63.43	0.094
**BMI (kg/m**^**2**^**)**	26.58	25.25	25.14	0.337	26.61	26.65	23.76	**0.007**
**WC (cm)**	80.72	78.27	79.22	0.736	80.32	82.20	75.82	0.102
**AEE (kJ/min)**	975.27	1063.51	1849.68	0.265	554.56	1498.53	1835.90	0.079
**Energy Intake (kJ)**	6397.32	8402.06	9881.90	** < 0.001**	8607.27	8136.98	8009.05	0.704
**Total cholesterol (mmol/L)**	5.64	5.79	5.78	0.813	5.59	5.69	5.94	0.362
**TG (mmol/L)**	1.32	1.47	1.50	0.679	1.40	1.42	1.47	0.958
**HDL (mmol/L)**	1.84	1.78	1.80	0.935	1.68	1.78	1.96	0.111
**LDL (mmol/L)**	3.20	3.33	3.29	0.871	3.27	3.25	3.31	0.974
	**Pattern 3**					**Pattern 4**		
	**T1 (low)**	**T2**	**T3 (high)**	***P*** **for trend**	**T1 (low)**	**T2**	**T3 (high)**	***P *for trend**
**Age (years)**	63.35	62.92	64.00	0.698	62.96	63.92	63.41	0.759
**Weight (kg)**	64.60	66.23	69.44	0.227	64.67	66.88	68.75	0.365
**BMI (kg/m**^**2**^**)**	24.51	25.76	26.64	0.140	25.14	25.65	26.14	0.652
**WC (cm)**	76.59	78.03	83.43	0.062	77.74	78.98	81.40	0.486
**AEE (kJ/min)**	1879.96	737.96	1292.31	0.159	816.65	1798.88	1294.29	0.259
**Energy Intake (kJ)**	8366.46	7591.91	8765.82	0.279	7050.09	8632.63	9031.26	**0.017**
**Total cholesterol (mmol/L)**	5.66	5.80	5.74	0.858	5.88	5.96	5.39	**0.049**
**TG (mmol/L)**	1.25	1.47	1.56	0.367	1.38	1.60	1.31	0.402
**HDL (mmol/L)**	1.90	1.76	1.77	0.552	1.83	1.72	1.87	0.538
**LDL (mmol/L)**	3.20	3.37	3.25	0.793	3.42	3.51	2.92	**0.032**

*BMI* Body mass index, *WC* Waist circumference, *AEE* Activity energy expenditure, *TG* Triglycerides

*HDL* High density lipoprotein, *LDL* Low density lipoprotein, *T1* Tertile 1, *T2* Tertile 2, *T3* Tertile 3 of the dietary pattern

The results of the correlational analysis are shown in Tables 3 and 4. It is apparent from Table 3 that there was a significant negative association between the selected nutrients and some of the inflammatory markers. The table shows negative correlations between the intake of dietary fibre, soluble and insoluble non-starch polysaccharides (NSP), vitamin C and niacin and with almost all the inflammatory markers. Vitamin D was negatively associated with IL-6 and CRP levels while Vitamin C was inversely correlated to IL-1β, IFN-α2, IFN-λ and IL-23. Niacin and folic acid were also negatively correlated with some of the inflammatory biomarkers as shown in Table 3 below. Sodium was however positively associated with MCP-1 while potassium had an inverse association. However, interleukin (IL)-10, IL-12p70, IL-17A and IL-18 were not significantly correlated with these nutrients.

**Table 3:** Correlations between intake of selected nutrients and inflammation markers (Whole group)

	**Fibre**	**Soluble NSP**	**Insoluble NSP**	**Vitamin D**	**Vitamin C**	**Niacin**	**Folic acid**	**Sodium**	**Potassium**
IL-1β (pg/ml)	-0.25*	-0.21	-0.27*	-0.02	-0.27*	-0.16	0.07	-0.03	-0.25*
IFN-α2 (pg/ml)	-0.33**	-0.25*	-0.36**	0.07	-0.22*	-0.27*	0.05	-0.12	-0.25*
IFN-λ (pg/ml)	-0.36**	-0.27*	-0.40**	-0.01	-0.23*	-0.24*	0.01	-0.01	-0.22
TNF-α (pg/ml)	-0.28*	-0.20	-0.32**	0.02	-0.10	-0.23*	0.04	0.10	-0.05
MCP-1 (pg/ml)	-0.10	-0.02	-0.14	-0.07	-0.05	-0.10	0.03	0.28*	-0.03
IL-6 (pg/ml)	-0.33**	-0.25*	-0.38**	-0.28*	-0.20	-0.26*	-0.25*	-0.07	-0.21
IL-8 (pg/ml)	-0.20	-0.11	-0.26*	0.02	-0.18	-0.13	0.05	0.01	-0.08
IL-10 (pg/ml)	-0.07	-0.04	-0.10	0.10	-0.09	-0.04	0.13	-0.01	-0.02
IL-12p70 (pg/ml)	0.02	0.05	-0.01	0.08	-0.06	-0.04	0.13	0.01	-0.01
IL-17A (pg/ml)	-0.03	-0.01	-0.09	0.06	-0.18	-0.07	0.10	-0.02	-0.03
IL-18 (pg/ml)	-0.08	-0.02	-0.08	0.01	-0.05	-0.04	0.02	0.08	-0.10
IL-23 (pg/ml)	-0.25*	-0.16	-0.30**	0.12	-0.36**	-0.06	0.16	-0.01	-0.15
IL-33 (pg/ml)	0.02	0.05	0.01	0.11	-0.10	0.06	0.16	0.19	0.05
CRP (µg/ml)	-0.02	-0.03	-0.01	-0.30**	-0.09	-0.23	-0.28*	-0.20	-0.16
Ferritin (µg/L)	-0.27*	-0.30**	-0.26*	0.02	-0.04	-0.11	-0.06	-0.17	-0.05

*NSP* Non-starch polysaccharides, *IL* Interleukin, *IFN* Interferon, *TNF* Tumour necrosis factor, *MCP-1* Monocyte chemoattractant protein – 1, *CRP* C-reactive protein

^*^P < 0.05

^**^P < 0.01

**Table 4:** Correlations between intake of selected foods and inflammation markers

	**Bread**	**Fruit**	**Vegetable**	**White meat**	**Red meat**	**Sauces/dressings**	**Yoghurt**	**Confectionery**	**Tea/coffee**
IL-1β (pg/ml)	0.05	-0.16	-0.20	0.19	-0.02	-0.06	-0.07	0.05	-0.13
IFN-α2 (pg/ml)	-0.05	-0.29*	-0.12	0.14	-0.08	0.02	-0.14	-0.01	-0.26*
IFN-λ (pg/ml)	-0.04	-0.31**	-0.19	0.19	-0.15	0.02	-0.21	0.03	-0.27*
TNF-α (pg/ml)	0.06	-0.19	-0.24*	0.08	-0.01	0.16	-0.09	0.18	-0.27*
MCP-1 (pg/ml)	-0.02	-0.16	0.07	0.09	0.04	0.03	-0.02	0.09	-0.15
IL-6 (pg/ml)	-0.06	-0.24*	-0.14	0.26*	-0.03	-0.04	-0.13	0.03	-0.23*
IL-8 (pg/ml)	0.13	-0.30**	0.09	0.21	-0.07	-0.08	-0.26*	0.15	-0.25*
IL-10 (pg/ml)	0.13	-0.05	-0.09	0.10	0.16	-0.01	-0.14	0.05	-0.10
IL-12p70 (pg/ml)	0.26*	0.07	0.05	-0.02	0.03	-0.28*	-0.29**	0.23*	-0.09
IL-17A (pg/ml)	0.10	-0.13	-0.01	-0.08	-0.07	-0.25*	-0.27*	0.18	-0.22
IL-18 (pg/ml)	-0.06	-0.17	0.046	-0.01	0.03	0.07	0.02	0.24*	0.01
IL-23 (pg/ml)	0.08	-0.36**	0.01	0.07	-0.01	-0.07	-0.19	0.07	-0.16
IL-33 (pg/ml)	0.18	0.04	-0.08	-0.08	0.11	-0.09	-0.18	0.28*	0.02
Ln CRP (µg/ml)	-0.19	-0.13	-0.34**	0.04	0.01	0.02	0.04	0.17	-0.02
Ln Ferritin (µg/L)	-0.02	-0.06	-0.05	0.31**	0.16	0.02	0.03	-0.07	-0.07

*NSP* Non-starch polysaccharides, *IL* Interleukin, *IFN* Interferon, *TNF* Tumour necrosis factor, *MCP-1* Monocyte chemoattractant protein – 1, *CRP* C-reactive protein

^*^P < 0.05

^**^P < 0.01

As shown in Table 4, intake of vegetables and especially fruits were negatively correlated with the inflammatory markers. Intake of yoghurt was also negatively associated with IL-8, IL-12p70 and IL-17A. Surprisingly, tea/coffee intake also showed a negative correlation with the cytokines. However, intake of confectionery was positively associated with the inflammatory markers.

Further multiple linear regression indicated a positive correlation which was observed between Pattern 2 and total cholesterol (TC) and HDL levels as shown in Table 5. Meanwhile, pattern 4 (meat and vegetables pattern) was however negatively correlated with TC, LDL and TC/HDL ratio.

**Table 5:** Multiple linear regression of the association between lipid profile and dietary patterns

Dependent variable	Independent variable	R^2^	β	Standard error	*P*-value
**Total cholesterol**		0.145*			
	Pattern 1		-0.067	0.098	0.538
	Pattern 2		0.250	0.099	**0.024**
	Pattern 3		0.173	0.099	0.115
	Pattern 4		-0.218	0.099	**0.047**
**Triglycerides**		0.023			
	Pattern 1		0.007	0.092	0.953
	Pattern 2		-0.071	0.092	0.539
	Pattern 3		0.133	0.092	0.254
	Pattern 4		-0.007	0.092	0.955
**HDL-cholesterol**		0.071			
	Pattern 1		0.034	0.055	0.763
	Pattern 2		0.226	0.055	**0.049**
	Pattern 3		-0.072	0.055	0.524
	Pattern 4		0.115	0.055	0.312
**LDL-cholesterol**		0.142*			
	Pattern 1		-0.090	0.095	0.411
	Pattern 2		0.162	0.095	0.140
	Pattern 3		0.162	0.096	0.139
	Pattern 4		-0.285	0.096	**0.010**
**Total/HDL Cholesterol ratio**		0.089			
	Pattern 1		-0.093	0.105	0.409
	Pattern 2		-0.090	0.105	0.423
	Pattern 3		0.134	0.105	0.235
	Pattern 4		-0.231	0.105	**0.043**

*β *Standardized coefficient, *HDL *High density lipoprotein cholesterol, *LDL *Low density lipoprotein cholesterol

*P > 0.05

## Discussion

This study is the first to assess the relationship between dietary patterns and an array of 15 inflammatory biomarkers. The results of this study show the importance of fruits, vegetables and dietary patterns that are healthy and nutrient-dense for lower circulating inflammatory markers and status. Owing to the fact that, chronic low-grade systemic inflammation is a risk factor in the development of chronic metabolic diseases, investigating the factors that contribute to high circulating inflammatory biomarkers is therefore important.

Pro-inflammatory cytokines are generally known for their degenerating and catabolic effects on tissue metabolism and homeostasis as well as their intracellular actions and are made up of mainly activated macrophages [19]. More so, in postmenopausal women especially, with the coupling effect of oestrogen deficiency; elevated levels of inflammatory cytokines have been linked to pain and many metabolic diseases [15], and these have been termed ‘inflammaging’ by researchers [20]. Increased production of pro-inflammatory cytokines such as IL-1β, IL-6, TNF-α and MCP-1 has also been linked to various types of cancer.

Most often anti-inflammatory cytokines exert opposite effects of pro-inflammatory cytokines by inhibiting the synthesis of these cytokines. However, both types of cytokines can work interactively in a spatial and dynamic network to create a balance of both the inhibitory and stimulatory effects [16, 19]. IL-10 is a known potent suppressor of pro-inflammatory cytokines while IL-6 is also considered an anti-inflammatory cytokine. Some cytokines in providing a syngenetic balance act a dual role in the regulation of the immune system. Cytokines such as IL-12 and IL-23 are considered as having a dual-role effect [21].

In this research, four dietary patterns were generated from the food groups. The four dietary pattens include pattern 1 (potato, bread, and fruit pattern), pattern 2 (soups and vegetables pattern), pattern 3 (fast-food pattern) and pattern 4 (meat and vegetables pattern) accounting for 14.5%, 13.5%, 12% and 8.7% of the variance explained respectively.

The results of this study show that dietary fibre and insoluble non-starch polysaccharides were inversely associated with IL-1β, IFN-α2, IFN-λ, TNF-α, IL-6, IL-23 and ferritin levels. This is in accordance with other studies which have reported that a high fibre diet is associated with a lower plasma level of IL-6 and TNF-α [22]. Intake of vitamin C and niacin were also inversely related to some of the inflammatory markers; this result is comparable to that of the study by Ellulu et al. 2015 which also indicated a reduction in inflammatory status on administration of vitamin C [23]. Vitamin C is known to work as an antioxidant capable of boosting the immune system.

Sodium intake was positively associated with monocyte chemoattractant protein-1 (MCP-1) which is in accordance with findings in the literature stating high salt intake increases MCP-1 synthesis [24, 25]. Meanwhile, intake of potassium was inversely correlated with IL-1β and IFN-α2 which may be due to the high consumption of fruits.

The result of this study is consistent with other studies showing a positive correlation between adherence to Western-style dietary pattern and CRP levels [26], this study indicated the Pattern 3 (fast food pattern) was positively associated with CRP. In addition, Muga et al. (2016) reported an inverse association between fruits and vegetables rich dietary pattern and CRP levels [27]. Likewise, three studies have also reported a negative correlation between fruits and CRP levels [7, 28, 29], this present study also indicates an adherence to vegetables and most especially fruits was negatively correlated with CRP, IFN-α2, IFN-λ, IL-6, IL-8, IL-23 and ferritin. This may be due to the high antioxidant/polyphenolic content of fruits. Our results therefore reiterate the significance of adequate consumption of fruit in order to decrease levels of circulating inflammation postmenopause. Surprisingly, inverse relationships were observed between tea/coffee and IFN-α2, IFN-λ, TNF-α, IL-6 and IL-8. This could be attributed to the polyphenolic content of tea/coffee intake amongst the women.

Pattern 2, which was characterized by high intake of soups and vegetables was positively correlated with TC and HDL-cholesterol. Pattern 4, which is characterized by high intake of meat and vegetables was inversely correlated with TC, LDL and total/HDL-cholesterol ratio. These results are similar to those of Lee and Kim 2018, that reported an inverse association between a prudent dietary pattern high in vegetables and fish and risk of low HDL cholesterolemia [30].

The strength of this study is in the ability to analyze and compare the relationship between diet and the various cytokines in postmenopausal women. However, the limitation of the study type lies in the fact that this is a cross-sectional study and the small sample size; therefore, more longitudinal and randomized controlled trials or studies are warranted to examine the effect of diet on these inflammatory biomarkers.

In conclusion, our findings reiterate the importance of dietary fibre, vitamin C, fruits, and vegetables in relation to the inflammation status in postmenopausal women. In addition, it also emphasizes the significance of dietary fibre, fruits, and vegetables in lowering ‘bad’ cholesterol (LDL). It is therefore important to investigate further beneficial dietary components of foods that can serve as a modulatory factor for the inflammation profile and cholesterol status in postmenopausal women. Further studies are needed to confirm the relationship and effect of the nutrients, foods, and dietary patterns on inflammation status.

## Data Availability

The datasets used and/or analysed for this study are available from the corresponding author on reasonable request.
